# Effect of heterogeneous substrate adhesivity of follower cells on speed and tension profile of leader cells in primary keratocyte collective cell migration

**DOI:** 10.1242/bio.058893

**Published:** 2022-03-08

**Authors:** Madhura Chakraborty, Baishali Mukherjee, Nanditha Nalinakshan, Arikta Biswas, Rajesh Kumble Nayak, Bidisha Sinha

**Affiliations:** 1 Department of Biological Sciences, Indian Institute of Science Education and Research Kolkata, Mohanpur, Nadia 741246, India; 2 Department of Physical Sciences, Indian Institute of Science Education and Research Kolkata, Mohanpur, Nadia 741246, India; 3 Center of Excellence in Space Sciences, India, Indian Institute of Science Education and Research Kolkata, Mohanpur, Nadia 741246, India

**Keywords:** Tension gradient, Membrane fluctuations, Adhesion, Collective cell migration

## Abstract

In single keratocyte motility, membrane tension is reported to be high at cell-fronts and believed to establish front coherence. To understand role of membrane mechanics in collective cell migration, we study membrane height fluctuations in cell sheets from fish scales using interference reflection microscopy (IRM). We report the monolayer to have cells lacking substrate adhesion and show that such ‘non-sticky’ cells can form bridges between leader cells and far-away follower cells. Do such interactions alter motility and membrane mechanics in such leaders? We find non-significant, but reduced speed for leaders with ‘non-sticky’ followers in comparison to other leaders. Cells show high phenotypic variability in their membrane fluctuation tension profiles. On average, this tension is found to be lower at cell fronts than the mid-section. However, leaders with non-sticky followers are more prone to display higher tension at their front and have a negative correlation between cell speed and front-mid tension difference. Thus, we conclude that intracellular tension gradients are heterogeneous in cell sheets and substrate adhesivity of followers can control the coupling of the gradient to cell speed.

## Abstract (precis)

**Summary:** Understanding how leading cells in keratocyte cell sheets are affected when their followers ‘ride’ on them and how this alters their basal membrane's height fluctuations and fluctuation tension.

## INTRODUCTION

Collective cell migration is central to processes like wound healing, development, and metastasis of cancerous cells. Mechanisms of motility vary from system to system (Ananthakrishnan and Ehrlicher, 2007); however, in single cells that use lamellipodia-driven motility, adhesion and membrane tension play crucial roles. The front-rear polarity is established by lipids and proteins implicated in responses to chemotactic signals; directed vesicle trafficking; regulation of actin nucleation, polymerization, filament-organization; and the regulation of integrin-based adhesion (Ridley et al., 2003). Such processes together impact the cell mechanically leading to coordinated movement of the front and retraction of the rear. As an outcome, not only is the tension of the plasma membrane and its distribution affected, tension also integrates the different inputs and forms the central mechanical regulator of motility. In cells like nematode sperm cells (Batchelder et al., 2011) and neutrophils (Houk et al., 2012), high tension enhances directional persistence while in isolated single keratocytes (Lieber et al., 2015), tension has a front-to-back gradient within the cell with high tension at the front. Optical-trap based experiments (Keren et al., 2008; Lieber et al., 2015) as well as theoretical modelling (Fogelson and Mogilner, 2014) attribute these intracellular tension-gradients to the continuous actin polymerization forces at the front and myosin-based contractile forces at the rear. How does the mechanical environment matter? In studies on keratocytes, interestingly, the actomyosin distribution was found to be less sharp in those keratocytes, which were not strictly isolated but tethered to the lagging cell-sheet (Svitkina et al., 1997). Moreover, even in single A2780 cells undergoing durotaxis (Hetmanski et al., 2019), the reported front-high tension gradient was lost when the substrate is uniformly rigid. Both examples underscore how differences in interactions felt at the front and the back of single cells affect its directionality and membrane tension gradient.

Like in single cell motility, collective cell migration also involves players like actin, Myosin II, integrins, proteolytic agents, cell–cell interactions and cell–matrix interactions among others (Ilina and Friedl, 2009). However, in contrast to the extensive studies about membrane mechanics in single cells, tension measurements have not been done in cell sheets displaying collective cell migration, to the best of our knowledge. How keratocytes that lead the cell sheets (leaders) and cells following leaders (followers) differ in their tension profiles from single isolated cells and how leader-follower interactions impact efficiency of collective migration, therefore, need thorough investigation.

We employ interference reflection microscopy (IRM; Biswas et al., 2017; Limozin and Sengupta, 2009) to image single cells in keratocyte cell sheets and quantify the temporal fluctuations in membrane-height of the basal plasma membrane from the glass-coverslips. These primary keratocytes emerge out of fish scales and move together as a front. Fluctuations are further used to derive effective ‘fluctuation tension’ (Shiba et al., 2016) of the membrane – a quantity that matches the ‘mechanical frame tension’ for over five decades of tension values (Fournier and Barbetta, 2008). It is to be noted that henceforth in this paper, the usage of the term ‘membrane tension’ pertain to measurements reported herein, would imply the fluctuation tension of the plasma membrane. Using this non-invasive method, we report details of adhesion to substrate and are able to not only measure but also map membrane fluctuations and tension during collective cell migration. Cell-sheets are known to move slower – expected as an outcome of cell–cell interactions. Do we expect the front tension to be actively maintained at a higher tension in such a situation? We, therefore, quantify the adhesion state of followers of the leaders and present the speed and tension profiles of these cells pooled separately depending on their interaction state.

## RESULTS AND DISCUSSION

### Heterogenous adhesion pattern in collective cell sheets of keratocytes

**Fig. 1.**
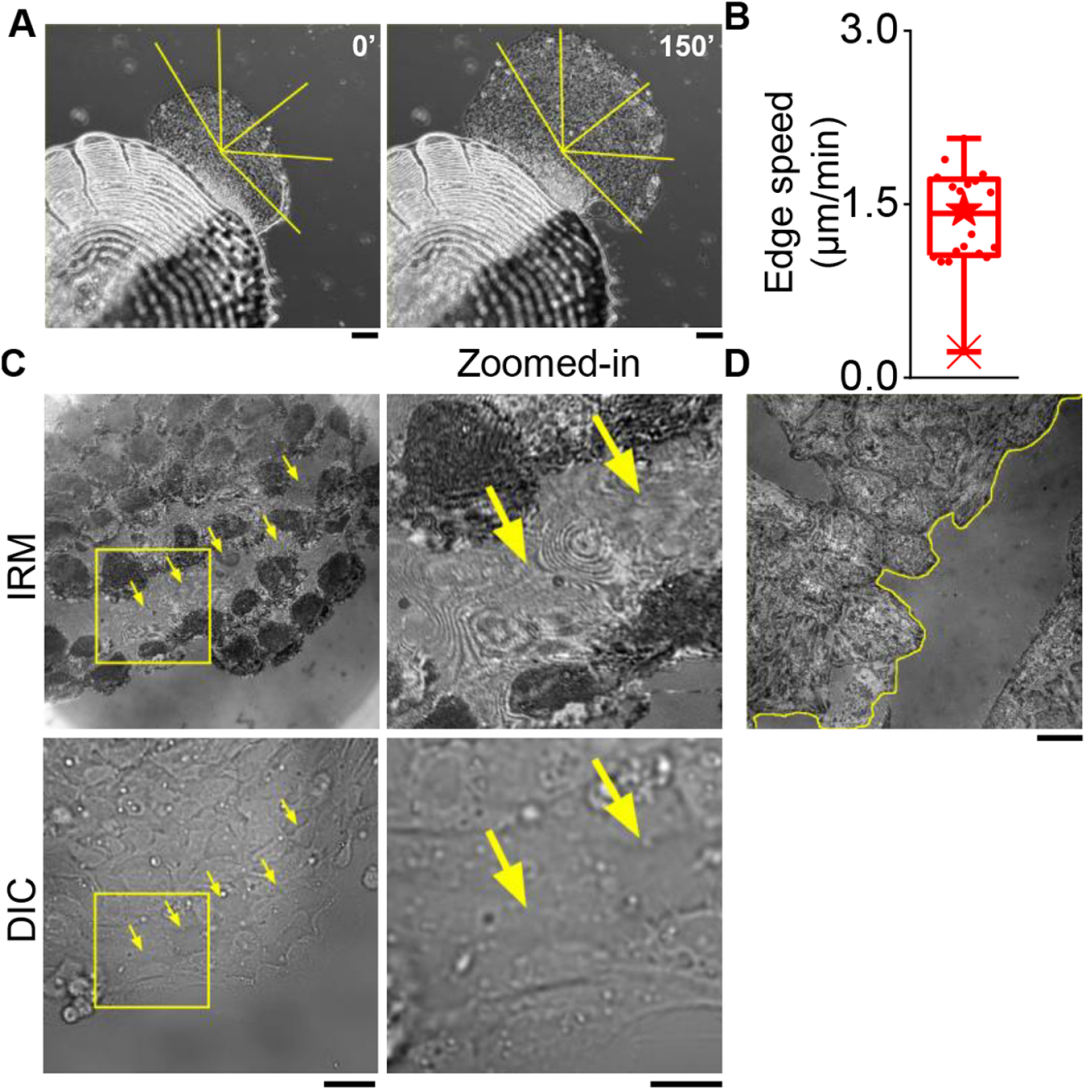
**Adhesion profile of keratocyte cell sheets from fish scales and HeLa monolayer.** (A) Representative images of increase in size of keratocyte cell sheet emerging out of fish scales. Scale bars: 100 µm. (B) Speed of expansion of the cell sheet as shown in A. (C) Left: IRM and corresponding DIC images of an edge of a cell sheet. Right: Zoomed-in views of an internal region as marked out in dashed yellow rectangle in C, left. Arrows indicate cells that show fringes in IRM but similar pattern as others in DIC. (D) IRM image of a HeLa monolayer with a wound marked in yellow dashed line. Scale bars: 10 µm. *N*_sheets_=4 from four independent experiments.

Cell sheets emerging out of fish scales within an hour of incubation, keep expanding in a fan-like shape (Movie 1; Fig. 1A; Fig. S1) with an edge speed of ∼1.45 µm/s (Fig. 1B; Fig. S1B). The whole front line of the sheet is composed of clear ‘leaders’ (Fig. S1A). While the sheet appears to be continuous in phase-contrast and DIC imaging, the adhesion status of cells can be best assayed by IRM. IRM shows adhered regions as dark pixels; as the distance of the membrane keeps increasing from the glass coverslip, the intensity increases till a maximum is reached at ∼100 nm, beyond which intensities start dropping again. Calibration with objects of known topology (∼60 µm diameter beads) is used for quantifying the conversion of intensity to relative height (distance of basal membrane from coverslip) (Biswas et al., 2017). Leaders show a dark footprint in IRM – indicating their strong although heterogenous attachment to the substrate (Fig. 1C). The followers display a previously unknown pattern of adhesion. Most followers well adhere to the substrate but for some, the cell is visible in DIC, but the attachment pattern is missing in IRM (Fig. 1C). This indicates that the cell is more than a micron away from the coverslip (no strong IRM signal) – but attached to its neighbors (visible in DIC). Such cells, therefore, appear to be ‘taking a ride’. Such a heterogeneous adhesion pattern is not observed in a monolayer of HeLa cells when wounded and left to repair (Fig. 1D).

### Cell bridges and multi-layered configuration

**Fig. 2.**
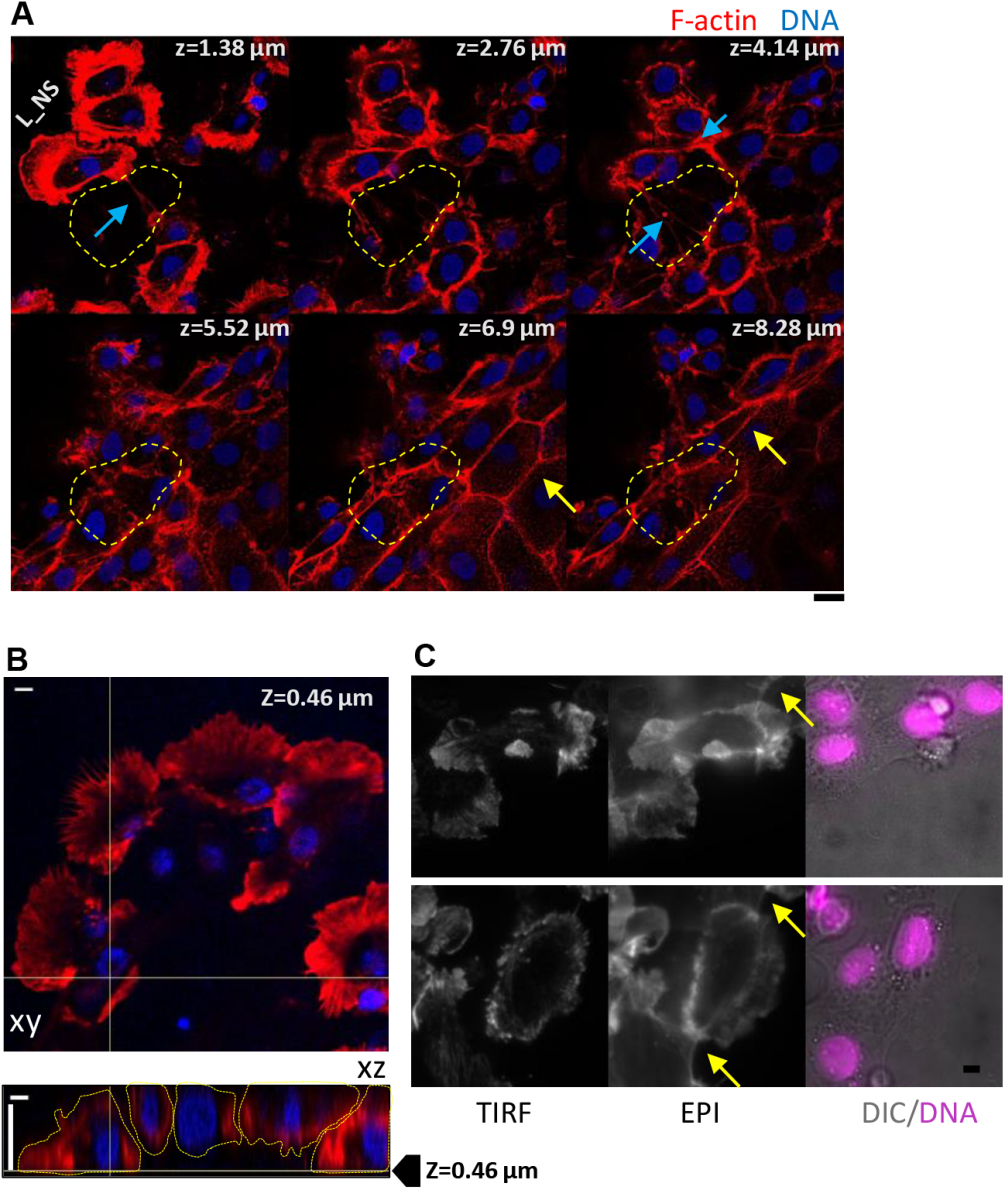
**Heterogeneous substrate adhesivity and cell brides.** (A) Representative selected z-slices of a 3D scan (z step=0.46 µm) of a section of cell sheet with the F-actin labelled with Phalloidin Alexa Fluor 568 (red) and DNA labelled with DAPI (blue). L_NS denotes the three leader cells that lack immediate followers. For the bottom-most cell, the space devoid of follower at lower z-planes (till 4.14 µm) is marked out with a dashed line. Blue arrows point out the cell–cell connections formed between cells spaces far away or at higher planes (only). Yellow arrows point to tight epithelial-like cell–cell connections even for cells that are not visible at lower planes. (B) Projections (top: xy, bottom: xz) of similar section as in A. XY projection is scanned at z=0.46 µm above glass and shows lack of followers for leaders moving towards top and left. XZ scan highlights formation of cell bridge. Cell outlines are guides to eye to follow the lack of substrate-attachment of central cells and proper cell–cell connections at higher z planes. (C) Representative images of F-actin labelled cells imaged at <100 nm depth using TIRF. Yellow arrows in epifluorescence image of same cells show concave edges of connected cells at higher z-planes. Scale bar: 5 µm. Scans are representative of 75 edges scanned from nine cell sheets in three independent experiments.

To further validate the attachment state of these cells we employ confocal microscopy of fixed, F-actin-labelled cell sheets (Fig. 2A,B). Following up the space (blue arrows, Fig. 2A) appearing cell-free at the basal planes, reveals the presence of cells at higher planes (yellow arrows). The cell outline overlaid on a representative xy and xz projection clearly establishes the presence of riders (Fig. 2B). Scanning through 75 edges of nine cell sheets in three independent tries, we observe 88% edges to have riders. Interestingly, besides single-cell-riders, we also find pluricellular structures (Vedula et al., 2014) or cell bridges (Fig. 2B), which are reported here for the first time for this system, to the best of our knowledge. In addition, we also provide evidence for a unique distribution of cell–cell interactions within cells. 3D scans reveal the epithelial-like cell–cell connection in cells at higher planes (Fig. 2A, yellow arrows) while retaining protrusive lamellipodial structures for migration at lower planes.

We also use total internal reflection fluorescence (TIRF) microscopy to further assess the adhesion state and F-actin distribution (Fig. 2C). TIRF is set at a penetration depth of <100 nm such that only cells within 100 nm of the coverslip fluoresces. When compared with the epi-fluorescence image captured at the same place the presence of cells at higher planes is clear. The presence of either actin-rich front-back fibers visible in confocal scans or concave edge (Fig. 2C, yellow arrows) of cell-bridges point to the existence of front-back forces on leader cells which are contributed by the riding cells and expected to be less in sections without riders.

In contrast to monolayers that are created by first ensuring attachment and then allowing growth, keratocyte cell sheets are formed from fish scales serving like a reservoir of cells. While this could potentially be the origin of the heterogeneous adhesion pattern, their presence is not essential for the maintenance of the adhesion profile. Our checks reveal that even when the scale is mechanically separated (Fig. S2), the sheet is left to incubate for hours (24–26 h) – the heterogeneous adhesion pattern persists.

We next investigate how cell bridges and gaps that are only at lower planes affect the mobility of leaders. We study how having followers that are adhered (termed ‘sticky’) or de-adhered (termed ‘non-sticky’) affect the movement of leader cells.

### Speed difference between leaders with sticky versus non-sticky followers

**Fig. 3.**
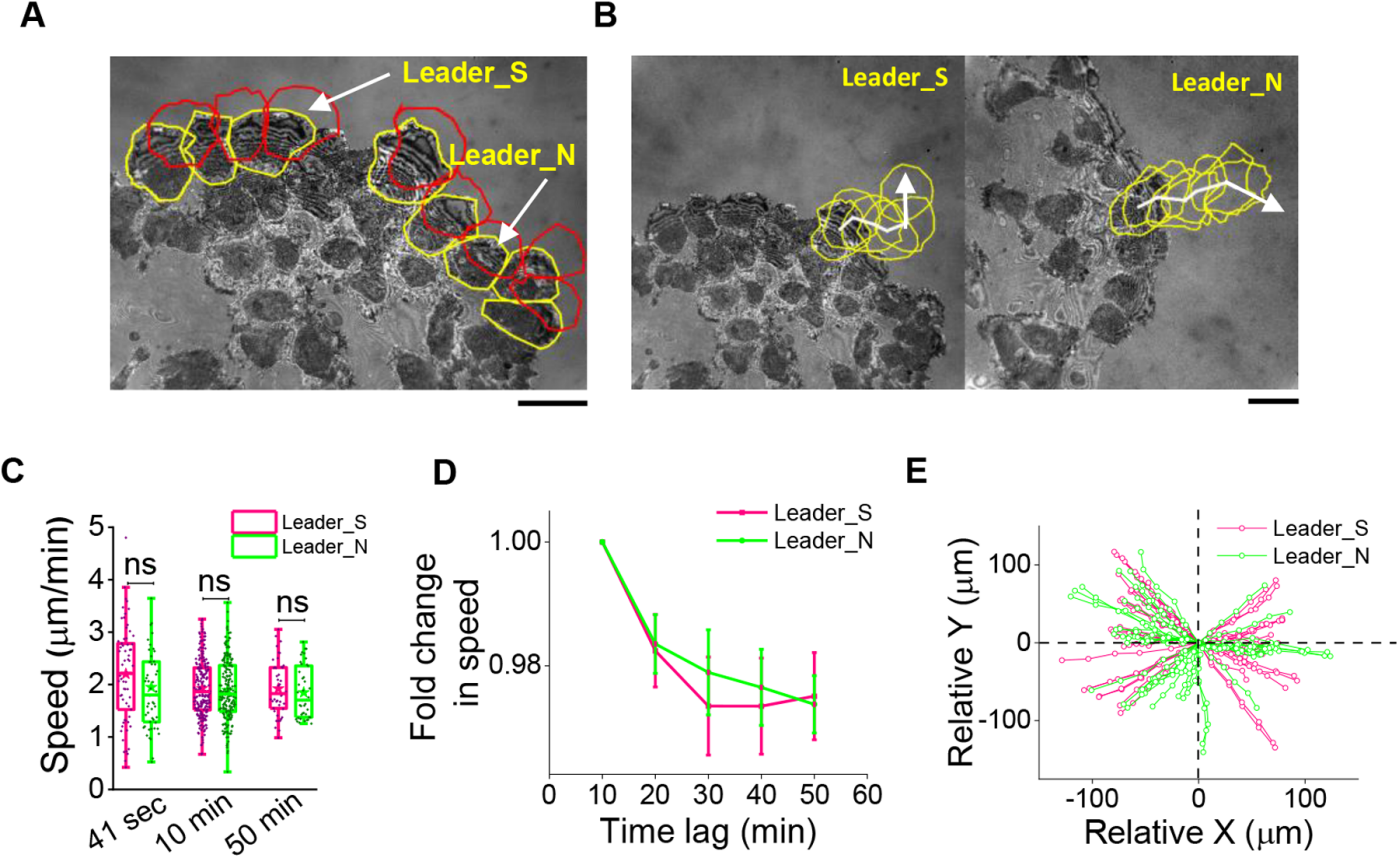
**Comparison of average speeds over different time-lags.** (A) Representative image of an edge of a cell sheet with yellow lines overlaid around leader cells whose immediate followers are well adhered to the substrate. Red lines outline positions of same cells after 10 min. (B) Outlining typical mobility of the two kinds of leader cells every 10 min for 50 min. (C) Speed calculated from time intervals of 41 s (left), 10 min (center) and 50 min (right). For time interval – 41 s: *N*_cells_=66, 53 for Leader_S and Leader_N, respectively, from seven independent experiments. For 10–50 min time intervals: *N*_cells_=67, 60 for Leader_S and Leader_N from eight independent experiments. (D) Fold change in speed calculated using different time-lags. Leader_N cells fall slower but the difference from Leader_S cells is statistically not significant. Trajectory plots of Leader_S and Leader_N cells (x vs y coordinate at different time points) when translated to (0,0). Statistical analysis was performed using Mann–Whitney *U*-test. ns denotes *P*-value>0.05. * denotes *P*-value<0.05. Scale bars: 10 µm.

Cell speeds was obtained from IRM images by tracking the centroid of the cells obtained from their outlines (Fig. 3A,B) either every 41 s or every 10–50 min (Fig. 3C). Based on the nature of attachment with the followers, leaders were classified as either Leader_S (followed by sticky or substrate-adhered followers) or Leader_N (followed by non-sticky followers). We report that when speed was measured by comparing centroids over 41 s or 10 min or 50 min intervals, no significant difference was found between the two types of leaders. However, when we calculate for single cells the change in speed measured as time-difference is increased, a reduction is found as expected for movers that have a diffusive component. The decay was faster for Leader_S pool indicating slightly less directionality (Fig. 3D); however, the difference is non-significant as also visible in the trajectory plots (Fig. 3E). Since tension gradients are also linked to motility, we next measure membrane tension gradients in these two kinds of leaders and their correlation with the short-term velocities.

### Fluctuations and tension ratio of front to mid part of single leader cells with sticky or non-sticky followers

Time-lapse IRM imaging (at 50 frames per sec for 2048 frames) reveals the dynamics of the adhesion profile, with bigger patterns remaining unchanged while smaller fluctuations are clearly captured (Movie 2). After calibration (see Materials and Methods), the amplitude of the temporal height fluctuations, SD_time_, is calculated from the time-lapse images and averaged over 6×6 pixel regions (∼432 nm×432 nm), which are specially selected (see Materials and Methods) and termed first branch regions (FBR). These regions are termed so because they are identified to fall in the first ∼100 nm of the coverslip corresponding to the first branch of the expected intensity-height pattern of the interference created in which the calibration used is applicable (Biswas et al., 2017). Effective tension is calculated from the power spectral density (PSD) of the fluctuations by considering contributions of the membrane's tension, confinement by the cytoskeleton/substrate, bending rigidity, effective temperature and the effective fluid viscosity around the membrane (Biswas et al., 2017; Biswas et al., 2019).

**Fig. 4.**
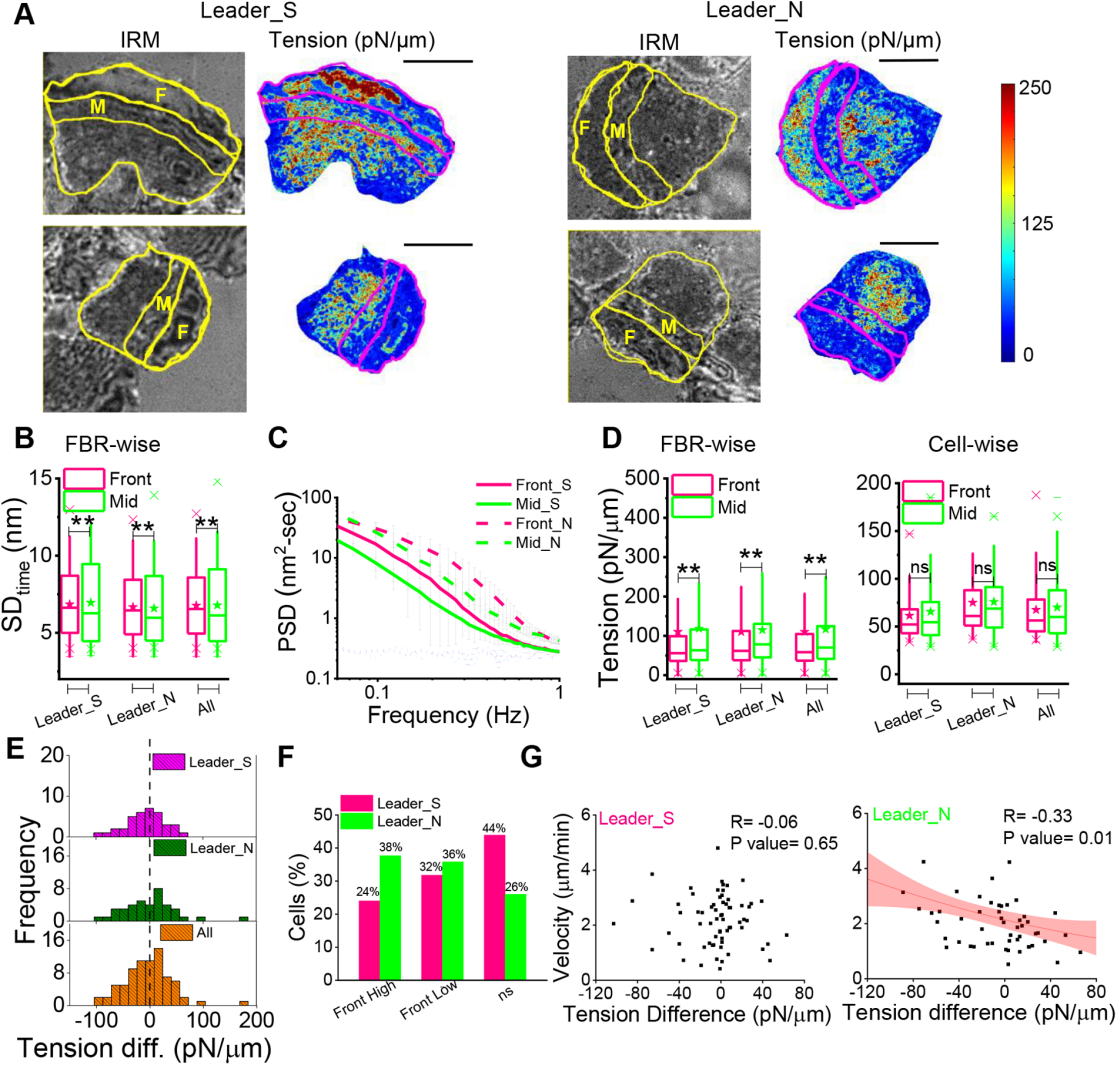
**Gradient of fluctuations and tension in leaders.** (A) Representative IRM images and corresponding tension maps of leader cells (Leader_S and Leader_N) with the front region and the middle region outlined and marked as F and M, respectively. (B) Comparison of SD_time_ between the front and mid FBRs among Leader_S, Leader_N and all leaders. Leader_S *N*_Front-FBRs_=33,654 *N*_Mid-FBRs_=24,285; *N*_cells_=66; Leader_N *N*_Front-FBRs_=27,381 *N*_Mid-FBRs_=20,253; *N*_cells_=53. (C) Average PSD of front- and mid-regions of leaders followed by sticky cells (Leader_S) and leaders followed by non-sticky cells (Leader_N); *N*_cells_=4; (D) Left: FBR wise comparison of tension of front and mid regions among multiple leaders and clubbed together (Leader_S front=15,440, mid=14,413; Leader_N front=11,624 mid=15,316 regions). Right: Cell wise comparison of tension of front and mid regions among multiple leaders and clubbed together (Leader_S=66, Leader_N=53 cells). (E) Distribution of tension difference between front- and mid-regions among the different population of leaders. (F) Correlation of tension difference between the front- and mid-part of each cell, with its speed calculated over a time interval of 41 s. The Pearson correlation coefficient (R) and *P*-value are mentioned. Sampling was done from seven independent experiments. Statistical analysis was performed using Mann–Whitney *U*-test and correlation test was performed using Pearson as well Spearman correlation method. ns denotes *P*-value>0.05, *P-value<0.05, **P-value<0.001. Scale bars: 10 μm.

Fluctuations are evaluated at the front of single cells covering 5 µm from the front edge and the middle section covering the next 5 µm (Fig. 4A). Tension maps reported from computational modeling of tension gradients (Fogelson and Mogilner, 2014) are used to decide the front-to-mid segregation with width of 5 µm to strike a balance between increasing sampling and constricting to narrow range of tension. Note that the rear of the cell is not reported here due to the presence of the nucleus in this region. We first show IRM images and corresponding tension maps of typical leader cells representing both types of tension gradient for each kind (Fig. 4A; Fig. S4). Tension maps are not restricted to pixels that are strictly within the usual ∼100 nm from the coverslip or the first branch. Hence, although they help in visualizing the tension distribution, we perform analysis at FBRs selected rigorously to compare the front and mid regions of both kinds of leader cells. Tension maps validate the choice of ∼5 µm as the width of the regions. The examples shown represent one front-high and one front-low tension gradient each for both the leader-types. Both leader types show enhanced fluctuations at their fronts (Fig. 4B). The ratio of front-to-mid SD_time_ when compared with 0 (by z-test): displayed significant increase from 0 (Leader_S *P*-value=0, Leader_N *P*-value=0) for both the pools. This indicates that temporal membrane flickering is enhanced at cell fronts. We also compare the level of damping of membrane fluctuations, captured by the parameter ‘exponent’ (Fig. S3D) whose absolute value reduces as the fluctuations are damped by restrictive surroundings (Brochard and Lennon, 1975; Gov et al., 2003; Biswas et al., 2017).

The PSDs (Fig. 4C) reveal differences between the front and mid regions which are further fitted with models (see Materials and Methods) to understand the mechanical parameters that drive the changes (Fig. 4D; Fig. S3). For both kinds of leaders, comparing FBRs pooled from the front or mid regions of all cells established that the front is maintained at lower tension (Fig. 4D). The difference in tension in single cells, however, is not significant, emphasizing the high regional and intercellular variability of front tension. Distribution of tension difference calculated for each cell also does not show any significant trend (Fig. 4E). However, we do find that Leader_N cells have a higher percentage of cells with front-high tension gradient than front-low or once with non-significant differences (Fig. 4F). Furthermore, unlike Leader_S cells, Leader_N cells display a significant negative correlation of the short-term speed and front-mid tension difference (Fig. 4G). This is in line with the mild reduction in speed and the higher propensity of front-high tension-gradient observed in Leader_N cells.

However, so far, we have only studied leaders. Do followers or cells not facing the edge but in inner layers have similarly heterogenous tension gradients?

### Followers usually display front-low tension gradient

**Fig. 5.**
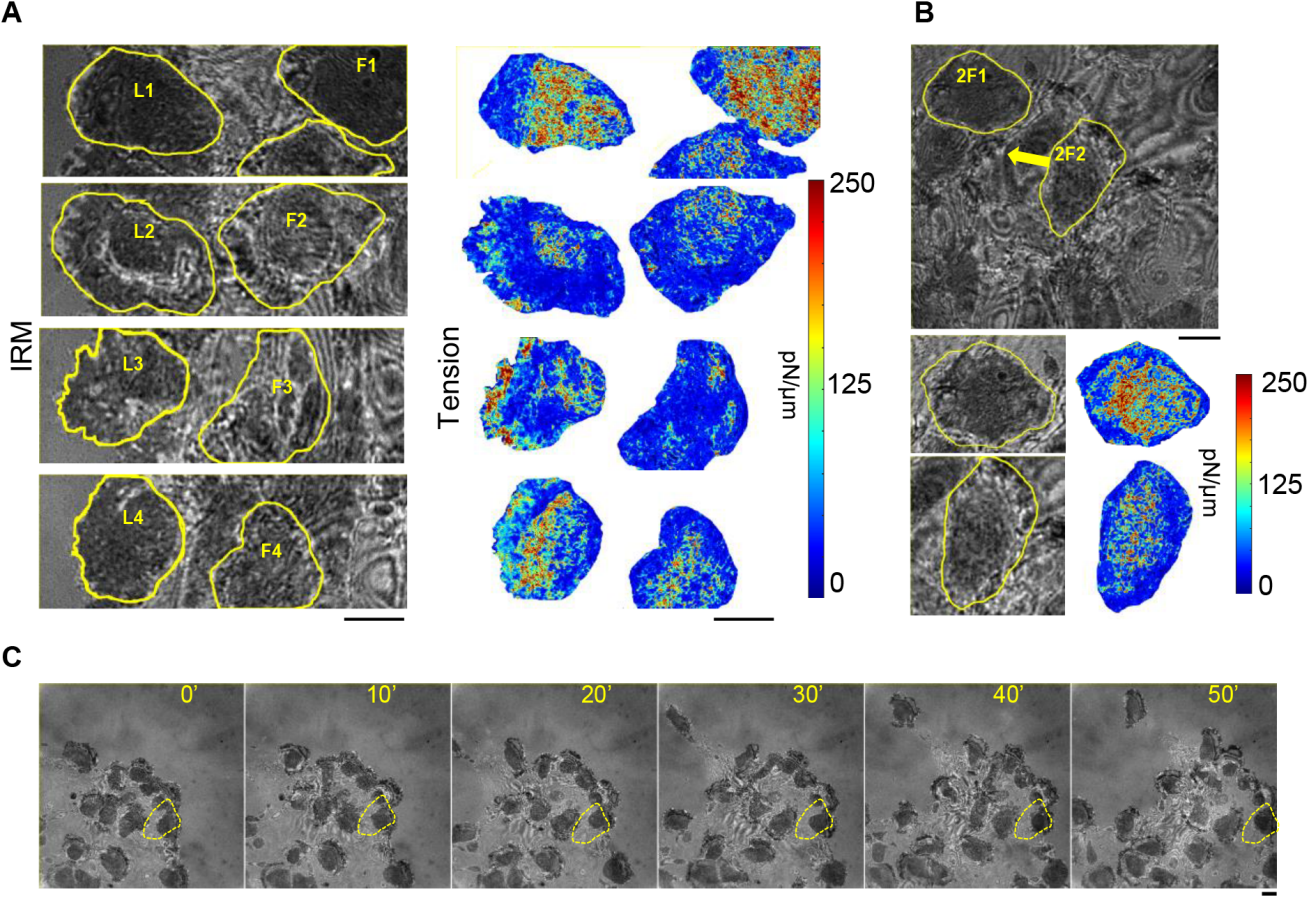
**Tension profile in deeper layers.** (A) Left: Representative IRM images of edge of a cell sheet with yellow outlines of leaders marked as L1-L4 and 1st layer of followers marked as F1-F4. Right: tension maps of the same. (B) Top: IRM image of two second layers of followers marked as 2F1 and 2F2 and the direction of migration denoted by yellow arrows. Bottom: Corresponding tension maps showing enhanced membrane tension in mid and rear portion. (C) Representative images of a cell edge followed for 50 min (representative of seven independent experiments) with yellow lines outline a follower that remains ‘sticky’ for the complete period. Scale bars: 10 μm.

Followers in the second line of cells (from the front) show front-low tension profile irrespective of the tension profile of the cell leading them (Fig. 5A). Followers in the third line of cells also show front-low tension profile (Fig. 5B) even if the cells following the followers are substrate-detached (Fig. 5C). We also show that for the 50 min period over which the sheet was followed; no switching of leader states could be observed and gap behind Leader_N moved along with the sheet. Leaders with non-sticky followers retained their identity as they moved (Fig. 5C).

## DISCUSSION

This work first establishes that keratocytes in cell sheets have highly patterned substrate-adhesivity. They retain sheet-continuity by maintaining strong cell–cell interactions at planes higher from the substrate while being weakly connected to each other at the basal plane. Even when clear epithelial-like polygonal cell shapes (Fig. 2A) are absent, relatively tight cell–cell connections were observed at higher planes (Fig. S6). Lack of substrate adhesion by followers appear as a “gap” at the back of Leader_N cells. Reports (Anon et al., 2012) have shown that lamellipodial cell spreading is the primary mode of gap closure in systems without cell damage with supracellular actin belts (observed usually at higher z planes in our system) helping in coordination of the movement of cells. Here, the Leader_N cells, however, do not move back but retain a forward velocity with the ‘gap’ also moving forward (see the vacant space outlined by the yellow line in Fig. 5C). Cues/coordination from the cell–cell connections at high planes hence are important. The space available at the substrate is not always enough to direct protrusive lamellipodia-based movement. The absence of large differences in speeds and front-mid tension gradients between Leader_N cells and other leaders is not surprising keeping in mind the fact that the sheet shows a continuous coherent front in the observed time window. However, the negative correlation found between cell speed and front-to-mid tension difference in Leader_N cells emphasizes the functional significance of the observed mild slowing down and preferred front-high tension in them.

The measurement of fluctuations and surface tension, and maps revealing heterogeneities are new and provide fresh insights. It is important to note that tension measurement is done at the lamellipodial regions but avoiding the sections protruding in the measurement time scales (41 s). While some cells display uniformly high-tension fronts with smooth edges (Fig. 4A, top right) we report a high phenotypic variability in the system (Fig. 4F). In single keratocyte studies have shown (Barnhart et al., 2017) cells to have different variable ‘front-smoothness’ and display protrusive waves with periods ranging from∼150–600 s. IRM enables us to follow protrusions every 20 ms and reveals protrusions that oscillate with a period ranging from 10 s and higher but can be asynchronous across different regions. Front movement and back retraction also can also asynchronous causing area fluctuations (Fig. S7). Together, with an expected slow flow of fluctuations (Biswas et al., 2019; Shi et al., 2018), they may explain the observed heterogeneity in tension. However, the negative correlation remains unclear and would need future investigations especially keeping in mind that traction and cell speed can have an inverse relationship (Doyle and Lee, 2005).

This study, thus, first shows how keratocyte cell sheets from cichlids offer an interesting platform in which new types of cell–cell interactions are present, and then, using them, advances the understanding of the nature and role of fluctuation tension distribution in collective cell migration.

## MATERIALS AND METHODS

### Fish and scale collection

Primary keratocyte cultures were collected from male/female adult Malawi golden cichlid (*Melanochromis auratus*) as per established protocols (Svitkina et al., 1997; Rapanan et al., 2014; Sun et al., 2016). Briefly, a fish was first anaesthetized with a 10% ethyl p-aminobenzoate for 3–5 min, before four to five scales were extracted. Scales were placed on glass-bottomed Petri dish with the inner side of the scale facing the surface. Small metal nuts placed on top of each scale were used to aid the attachment. Within 5 min, 2 ml of growth media was added in the dish and incubated at lab temperature (around 23°C) for 3–4 h unless otherwise specified. Growth media consisted of Leibovitz-15 (L-15) media (Gibco, Thermo Fisher Scientific, USA) supplemented with 15 mM HEPES buffer (Sigma-Aldrich), 10% FBS (Fetal Bovine Serum) (Gibco, Thermo Fisher Scientific) and 1% Anti-anti (Antibacterial-antimicrobial) (Thermo Fisher Scientific). The final pH adjusted to around 7.3 (Sun et al., 2016). HeLa cells (ATCC, CCL-2) were grown in growth media composed of Dulbecco's Modified Essential Medium (DMEM, Gibco, Life Technologies, USA), 10% FBS (Gibco, HI, USA) and 1% anti-anti (Gibco). Cells were checked for mycoplasma and were negative. Approval from the Institute Bio Safety Committee had been obtained for the HeLa cell line used.

### Cell fixation and F actin labelling

Fixation of keratocyte cells was performed using 3% paraformaldehyde (PFA) for 15 min at 37°C followed by washing once with PBS. 0.2% Triton-x-100 was added on the fixed cells and incubated for 2 min for permeabilization which further followed by another wash with PBS. F actin labelling was performed by incubating fixed cells with 1:200 dilution (33 nM) of Phalloidin Alexa Fluor 568 (Invitrogen, Thermo Fisher Scientific) for 1 h in dark. 50 ng/ml DAPI (Sigma-Aldrich) was used along with Phalloidin to label DNA. Imaging was done in 2 ml of PBS followed by staining.

### Imaging

Olympus IX81 microscope (Olympus, Japan) equipped with CMOS camera was used for capturing DIC/Phase contrast images of cell sheet at different magnifications (10x, 40x, 60x) for visualizing migration.

For IRM imaging, Nikon Eclipse Ti-E motorized inverted microscope (Nikon, Japan) was used. Cells were imaged under 60x Plan-Apo (water immersion, NA 1.22) with an external 1.5x magnification objective with a CMOS camera (ORCA-Flash 4.0, Hamamatsu, Japan). A 100 W mercury arc lamp, an (546±12 nm) interference filter and a 50-50 beam splitter were used, and fast time-lapse imaging was performed at 50 frames/s for 2048 frames.

Confocal imaging was done on Laser scanning confocal microscope (Carl Zeiss, LSM 710) with 100× oil objective lens (NA 1.46). Z-stack images of keratocyte cell sheets were taken with a step size of 460 nm (1 pixel=83 nm).

For TIRF, Olympus IX-83 inverted microscope (Olympus, Melville, USA) equipped with a 100X NA 1.49 oil immersion TIRF objective (PlanApo, Olympus) was used. Images were acquired using CMOS camera (ORCA Flash 4.0 Hamamatsu, Japan). A 561 nm laser beam was used as laser source for TIRF. All images were taken at 200 ms exposure time and 75 nm penetration depth.

### Calibration for IRM

For converting IRM image to membrane topology (height of basal membrane from glass coverslip) four steps were undertaken. First, stuck 60 µm diameter polystyrene beads (Bangs Laboratories) were imaged at various exposure times. Secondly, the radial intensity profiles were used to calculate the conversion factor for intensity difference (ΔI, au) to height (Δh, nm) conversion for the whole range of contrast obtained in the images. Contrast of an interference image is characterized by the maximum and the minimum or background intensity. Altering contrast mimics the effect of altering reflectivity. For a particular conversion factor to be applicable on cells, the regions must fall in the first branch with a membrane-height ranging from 0–100 nm and the contrast of the interference image must match with the bead-image used to calculate the factor. The third step thus involved measuring the contrast of the images of cells and using the interpolated bead-derived data to estimate the conversion factor for the particular contrast. Finally, the conversion factor was applied to first branch pixels which were selected by checking if they could be spatially connected to a first minima without passing through a first maxima (Biswas et al., 2017).

### Analysis of edge and cell speed

Sheet speed was calculated by first getting kymographs of cell sheets across radial lines and extracting edge speed as the distance covered by the expanding edge per unit time. Cell velocities were calculated by drawing the outline of the cells, finding centroids and finding distance between centroids per unit time.

### Analysis of fluctuations

The first part of the analysis characterizes the amplitude of the temporal fluctuations at particular pixels and termed SD_time_. It is the average standard deviation of the relative height at any particular pixel with the averaging done over a 432×432 nm^2^ region comprising of 6×6 pixels. The second part of the analysis characterizes the distribution of the fluctuation amplitude (or power) across different frequencies. The PSD of fluctuation signal is plotted as log (PSD) versus log (Frequency). The slope between 0.04–0.4 Hz of the log(PSD)-log(frequency) plot is fitted to a straight line, and defined as exponent. All the mechanical parameters were derived from fitting the PSDs of FBRs to an equation:

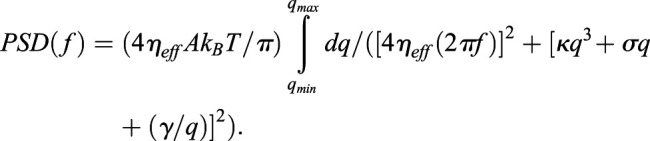
The value of bending rigidity (κ) here fixed as 15 k_B_T. Here η_eff_ denotes effective cytoplasmic viscosity, A denotes active temperature, σ is membrane tension and γ is confinement. MATLAB (Mathworks) was used for analysis and Origin (OriginLab Corporation). Parameters (mean, median, s.d., number of samples, *P*-values) are listed in Tables S1 and S2.

## Supplementary Material

Supplementary information
