# Supplementary material for: Effect of heterogeneous substrate adhesivity of follower cells on speed and tension profile of leader cells in primary keratocyte collective cell migration
Source: Biol Open. 2022 Mar 8;11(3):bio058893. doi: 10.1242/bio.058893 (PMC8918985; doi:10.1242/bio.058893)
Supplement: Supplementary information [file biolopen-11-058893-s1.pdf]

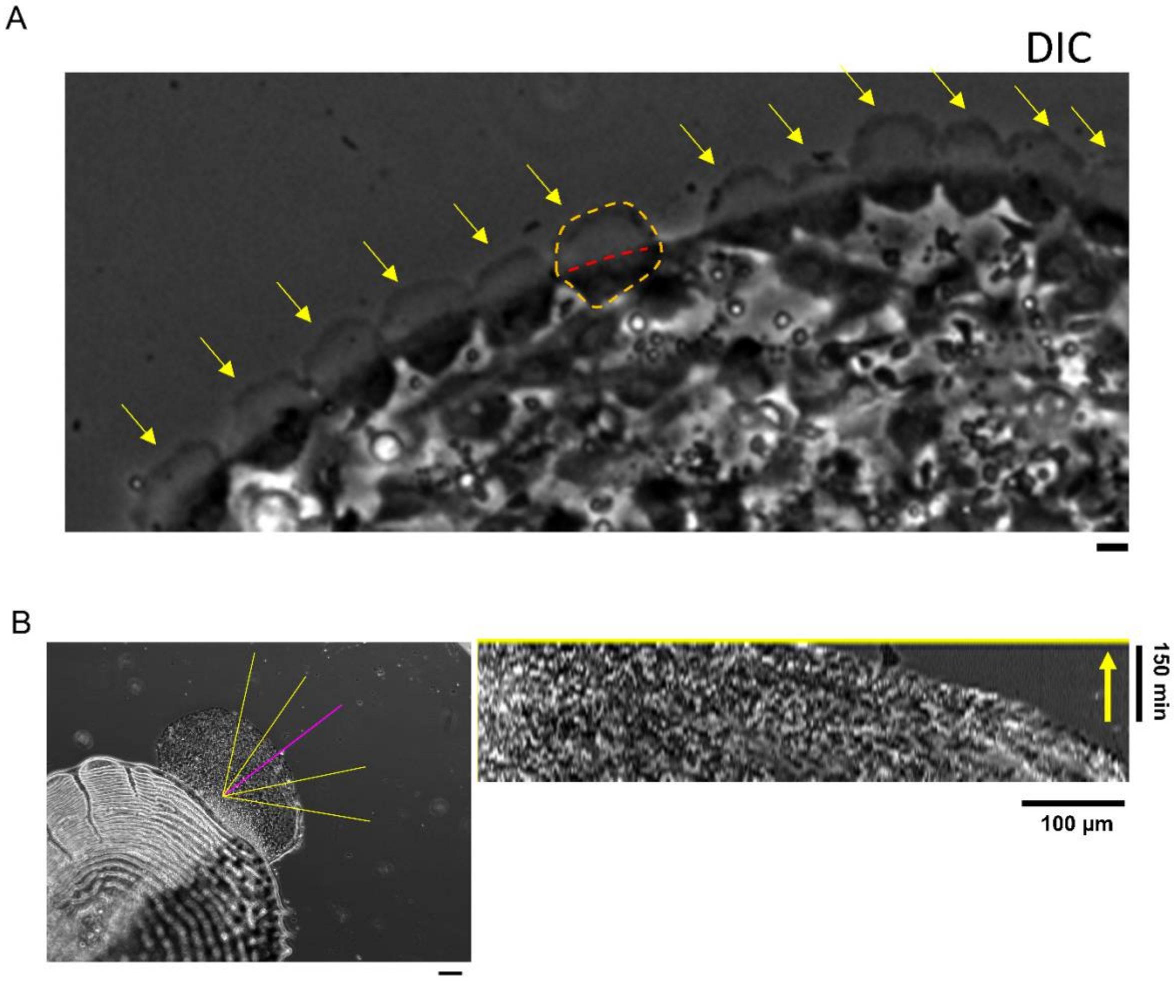

Fig. S1. Edge velocity calculation A) Representative DIC image of a typical keratocyte sheet showing all the leaders denoted by yellow arrows and a typical line like pattern at the mid region marked by magenta dotted structure. B) Left: Cells moving out from under a scale, with lines in yellow drawn to analyse from kymographs to measure velocity along those ROIs. Right: A typical kymograph (calculated from pink line ROI displayed in B) showing edge velocity of a cell sheet. Scale bar = 100 µm. Yellow arrow indicates direction of time.

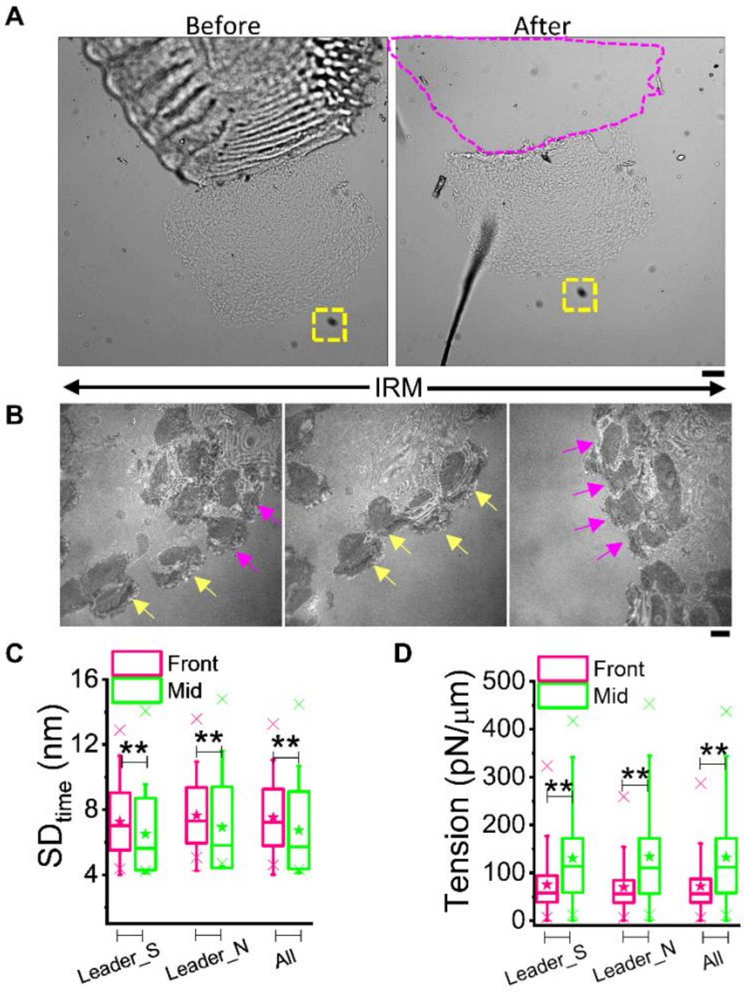

Fig. S2. Cell sheet after mechanical isolation of scale. (A) DIC images of cell sheet before (left) and after removal of scale (right), pink dashed outline denotes the old position of scale and yellow boxes are drawn around particular debris on the coverslip to highlight the same area. Scale bar = 100 µm. (B) IRM images of leaders with substrate-adhered followers pointed out by pink arrows (Leader_S), leaders with substrate-deadhered followers pointed by yellow arrows (Leader_N),scale bar= 10 µm (C) FBR wise comparison of temporal fluctuations between front and mid regions of Leader_S and Leader_N and clubbed together (nFBRs: Leader_S front: 7565, mid: 5509; Leader_N front: 14008, mid: 7191) (D) FBR wise comparison of membrane tension between front and mid regions of Leader_S and Leader_N and clubbed together (nFBRs: Leader_S front: 4486, mid: 3510; Leader_N front: 8610, mid: 4677) Leader_S: Ncells = 13, Leader_N: Ncells = 17. Statistical analysis was performed using Mann-Whitney U test. ** denotes p value <0.001.

Biology Open • Supplementary information

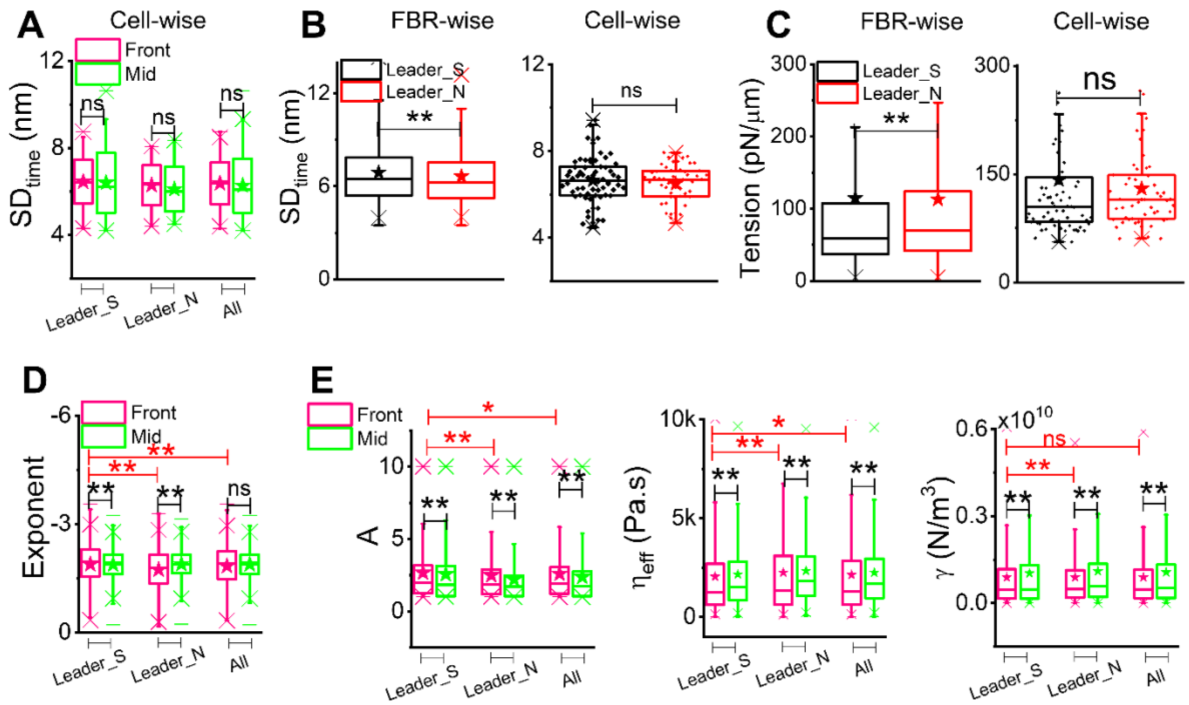

Fig. S3. Box plot comparison of different mechanical parameters A) cell wise comparison of SDtime between front and mid parts of leader cells (Leader_S: Ncells = 66, Leader_N: Ncells = 53 B) (left) FBR wise comparison of SDtime between Leader_S and Leader_N cells with front and mid clubbed together (Leader_S: nFBRs = 57939; Leader_N: nFBRs = 47634) (right) cell wise comparison of SDtime with front and mid clubbed together (Leader_S: Ncells = 66, Leader_N: Ncells = 53) C) (left) FBR wise comparison of membrane tension between two different pool of ledaers with front and mid clubbed together (nFBRs: Leader_S: 29853, Leader_N: 26940) (right) cell wise comparison of the same (Leader_S: Ncells = 66, Leader_N: Ncells = 53) D) FBR wise comparison of exponent between front and mid among different pool of leaders and all leaders clubbed together (nFBRs : Leader_S: front: 13373, mid: 981; Leader_N front: 6580, mid: 5345) E) (left) FBR wise comparison of active temperature between front and mid among different pool of leaders and all leaders clubbed together (centre) FBR wise comparison of effective cytoplasmic viscosity between front and mid among different pool of leaders and all leaders clubbed together (right) FBR wise comparison of confinement between front and mid among different pool of leaders and all leaders clubbed together (nFBRs: Leader_S front: 15440, mid: 14413, Leader_N front: 11624, mid: 15316) Statistical analysis was performed using Mann-Whitney U test. ns denotes p value >0.05. * denotes p value < 0.05 ** denotes p value <0.001.

Biology Open • Supplementary information

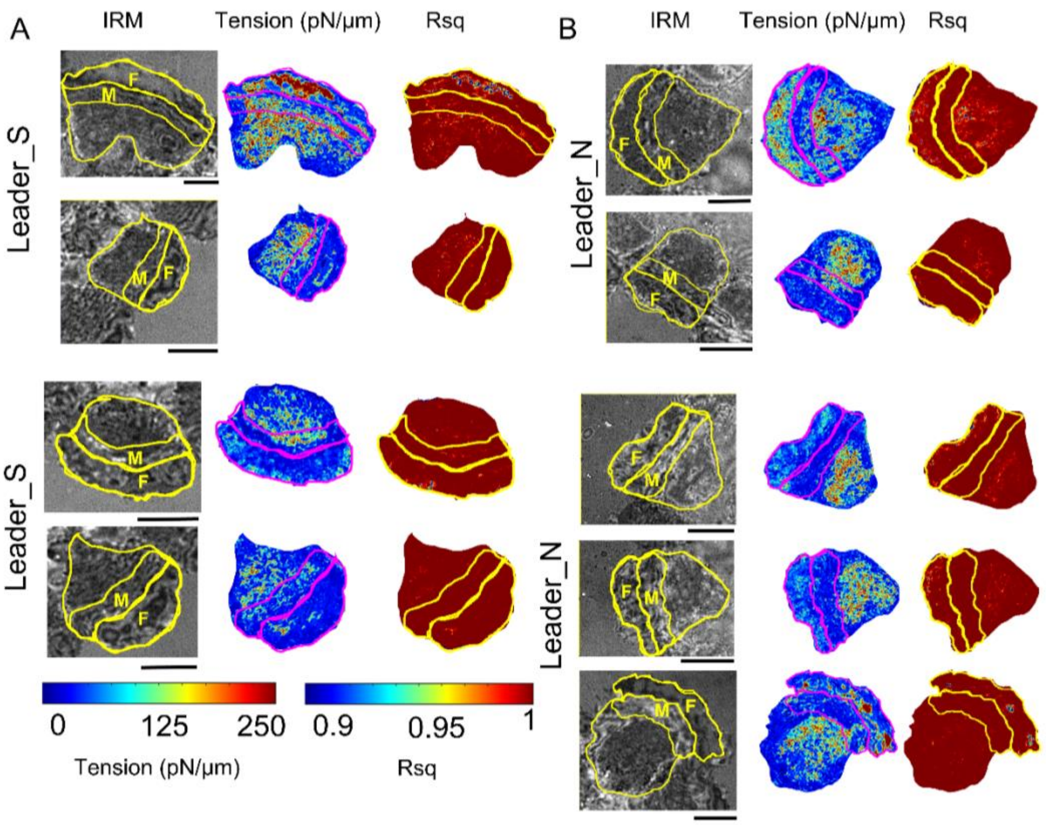

Fig. S4. Tension and their corresponding R^2 (Rsq) maps of leaders A) (left column) Leader_S with front and mid regions marked as F and M (middle column) respective tension maps, front and mid marked with magenta lines (right column) R^2 maps, front and mid marked with yellow lines B) (left column) Leader_N with front and mid regions marked as F and M (middle column) respective tension maps, front and mid marked with magenta lines (right column) R^2 maps, front and mid marked with yellow lines. Scale bar = 10 µm.

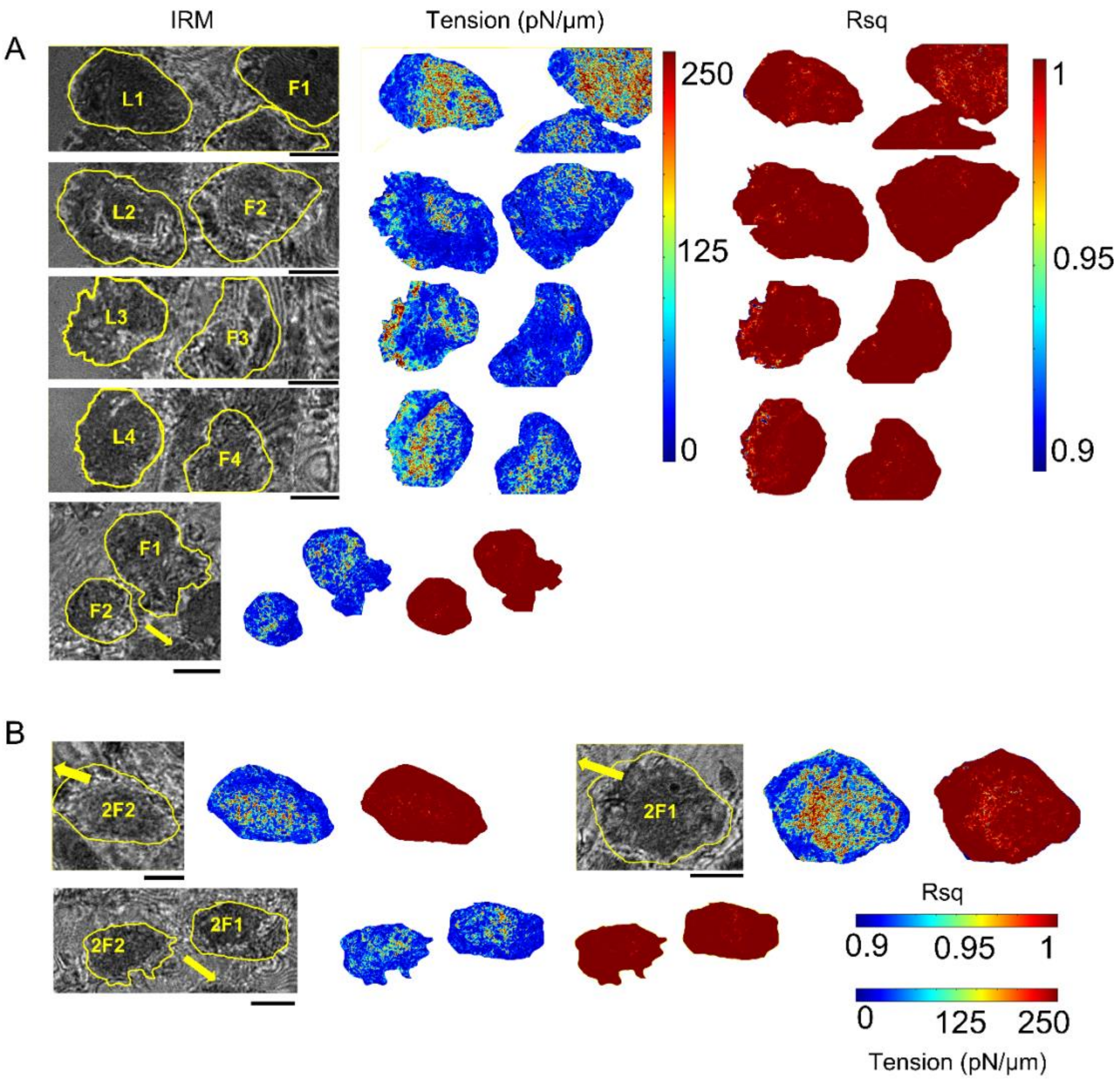

Fig. S5. Tension maps and corresponding R^2 (Rsq) maps of leaders and followers A) (left panel) (top to bottom) IRM images of leaders with first layer of followers marked as L1-L4 and F1-F4 respectively (middle panel) tension maps of leaders with 1st followers (right panel) R^2 maps of the same B) (top and bottom) IRM images of 2nd layer (2F) of followers with their respective tension and R^2 maps beside them respectively, direction of migration marked with yellow arrows. Scale bar = 10 µm.

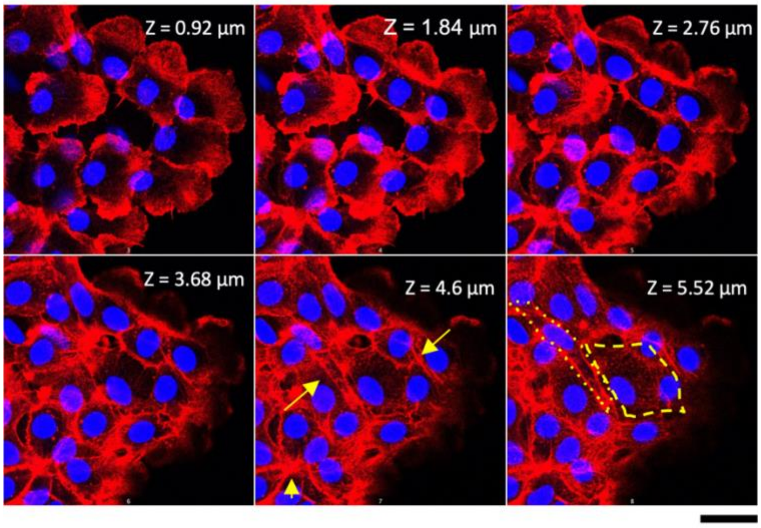

Fig. S6. Tight coupling of cells at higher z-planes. Representative confocal z-slices of a cell sheet with F-actin labelled with Phalloidin Alexa Fluor 568 and DNA with DAPI. Cells are either not attached or less tightly attached with each other at lower planes however are strongly attached at higher z-planes even if sharp-edged hexagonal packing (as in Fig 2A) is not observed. Yellow dashed line at z = 5.52 µm depicts the presence of a stretched-out cell over a gap at z = 0.92 µm. The yellow dotted line highlights a highly straightened cell visible only at a high z-planes (z > 4.6 µm). Scale bar = 10 µm.

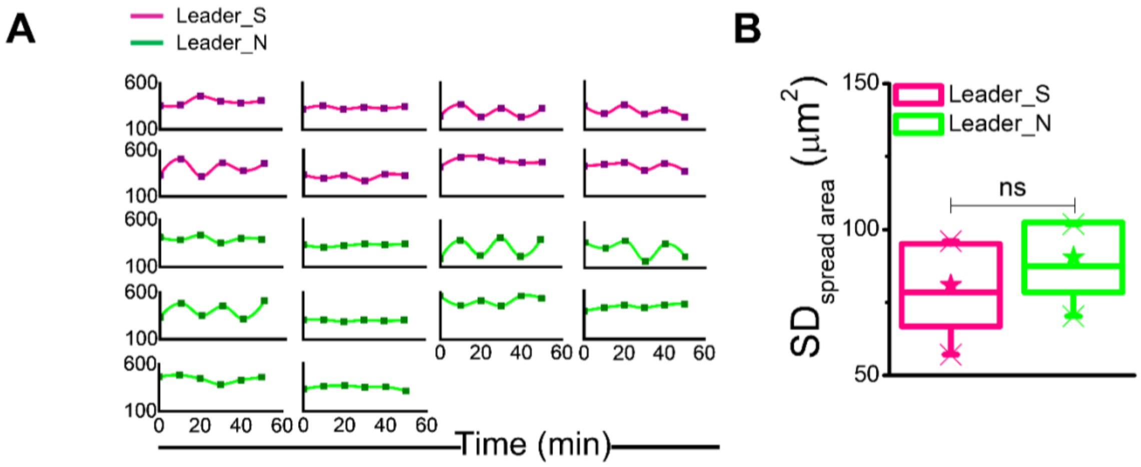

Fig. S7. (A) Fluctuations in cell spread area of individual cells during migration. (B)Variability (standard deviation) in cell spread area measured over 50 min.

Biology Open • Supplementary information

Table S1. **Descriptive statistics for all parameters plotted in the main figures.** Ncells denote the number of cells and nrois or nFBRs denote the number of ROIs (region of interests) or FBRs respectively used for the corresponding figure.

Figure 1B								
**Parameters**	**Condition**	**N**	**n_{rois}**	**Mean**	**SD**	**SEM**	**Median**	
Edge speed (µm/min)	Growth media	4	20	1	0.4	0.1	0.9	
**Figure 3C**								
**Parameters**	**Condition**	**N_{cells}**	**n**	**Mean**	**SD**	**SEM**	**Median**	**p-values (wrt Leader_S)**
Speed (µm/min)	Leader_S (41 sec)	66	66	2.19	0.92	0.11	2.21	
	Leader_N (41 sec)	53	53	1.95	0.84	0.11	1.8	0.0935
	Leader_S (10 min)	67	335	2.03	0.54	0.07	1.87	
	Leader_N (10 min)	60	300	1.89	0.48	0.06	1.76	0.14808
	Leader_S (50 min)	67	46	1.99	0.56	0.07	1.83	
	Leader_N (50 min)	60	42	1.84	0.49	0.06	1.69	0.11486
**Figure 4B**								
**Parameters**	**Condition**	**N_{cells}**	**n_{FBRs}**	**Mean**	**SD**	**SEM**	**Median**	**p-values (wrt Front)**
SD_{time} (nm)	Front; Leader_S	66	33654	6.8	1.8	0.01	6.6	
	Mid; Leader_S	66	24285	6.9	2.5	0.02	6.3	0
	Front; Leader_N	53	27381	6.7	1.8	0.01	6.4	
	Mid; Leader_N	53	20253	6.6	2	0.01	5.9	0
	Front; All	119	61035	6.8	1.8	0.007	6.5	
	Mid; All	119	44538	6.8	2.3	0.01	6.1	0
**Figure 4D**								
**Parameters**	**Condition**	**N_{cells}**	**n_{FBRs}**	**Mean**	**SD**	**SEM**	**Median**	**p-values (wrt Front)**
Tension (pN/µm) FBR wise	Front; Leader_S	66	15440	110	311.8	2.5	55.9	

Biology Open • Supplementary information

	Mid; Leader_S	66	14413	118	325.3	2.7	63.3	6E-31
	Front; Leader_N	53	11624	110	238.3	2.2	61.8	
	Mid; Leader_N	53	15316	115	198	1.6	78.3	4E-78
	Front; All	119	27064	110	282.6	1.7	58.5	
	Mid; All	119	29729	117	267.4	1.6	70.2	3E-118
**Parameters**	**Condition**	**N_{cells}**	**n_{FBRs}**	**Mean**	**SD**	**SEM**	**Median**	**p-values (wrt Front)**
Tension (pN/µm) Cell wise	Front; Leader_S	66		61.4	26.7	3.3	52.1	
	Mid; Leader_S	66		65.5	34.5	4.3	54.5	0.94738
	Front; Leader_N	53		75.1	41.6	5.7	61	
	Mid; Leader_N	53		75.9	34.5	4.7	68.8	0.61987
	Front; All	119		67.5	34.7	3.2	56.4	
	Mid; All	119		70.1	34.8	3.2	60	0.76822

Biology Open • Supplementary information

Table S2. Descriptive statistics for all parameters plotted in supplementary figures. N_{cells} denote the number of cells and n_{rois} or n_{FBRs} denote the number of ROIs (region of interests) or FBRs respectively used for the corresponding figure.

Figure S3A								
**Parameters**	**Condition**	**N_{cells}**	**n_{FBRs}**	**Mean**	**SD**	**SEM**	**Median**	**p-value (wrt Front)**
SD_{time} (nm)	Front; Leader_S	66	66	6.44	1	0.123	6.41	
	Mid; Leader_S	66	66	6.39	1.39	0.171	6.2	0.47341
	Front; Leader_N	53	53	6.29	0.91	0.126	6.32	
	Mid; Leader_N	53	53	6.10	1.03	0.142	5.99	0.258
	Front; All	119	119	6.37	0.97	0.089	6.38	
	Mid; All	119	119	6.27	1.25	0.114	6.07	0.18587
**Figure S3B**								
**Parameters**	**Condition**	**N_{cells}**	**n_{FBRs}**	**Mean**	**SD**	**SEM**	**Median**	**p-value (wrt Front)**
SD_{time} (nm)	Leader_S	66	57939	6.89	2.14	0.009	6.49	
	Leader_N	53	47634	6.63	1.92	0.009	6.26	0
	Leader_S	66	66	6.64	1.10	0.136	6.64	
	Leader_N	53	53	6.49	0.84	0.116	6.68	0.60588
**Figure S3C**								
**Parameters**	**Condition**	**N_{cells}**	**n_{FBRs}**	**Mean**	**SD**	**SEM**	**Median**	**p-values (wrt Front)**
Tension (pN/µm)	Leader_ S	66	29853	114.3	318.4	1.843	59.30	
	Leader_ N	53	26940	112.9	216.3	1.318	70.06	8E-99
	Leader_ S	66	66	141.9	102.7	12.642	105.10	
	Leader_ N	53	53	129.8	56.8	7.803	114.83	0.43345

Figure S3D								
**Parameters**	**Condition**	**N_{cells}**	**n_{FBRs}**	**Mean**	**SD**	**SEM**	**Median**	**p-value (wrt Front)**
Exponent	Front; Leader_S	38	1337 3	-1.89	0.58	0.005	-1.96	
	Mid; Leader_S	38	9819	-1.88	0.4	0.004	-1.88	5E-14
	Front; Leader_N	21	6580	-1.74	0.57	0.007	-1.8	
	Mid; Leader_N	21	5345	-1.89	0.39	0.005	-1.9	2E-30
	Front; All	59	1995 3	-1.84	0.58	0.004	-1.91	
	Mid; All	59	1516 4	-1.88	0.4	0.003	-1.89	0.23452
**Figure S3E**								
**Parameters**	**Condition**	**N_{cells}**	**n_{FBRs}**	**Mean**	**SD**	**SEM**	**Media n**	**p-value (wrt Front)**
A	Front; Leader_S	66	1544 0	2.6	2.8	0.01	2	
	Mid; Leader_S	66	1441 3	2.7	2.6	0.02	1.8	0
	Front; Leader_N	53	1162 4	3.2	2.3	0.02	1.9	
	Mid; Leader_N	53	1531 6	2.6	2.2	0.01	1.7	0
	Front; All	119	2706 4	3.2	2.6	0.01	1.9	
	Mid; All	119	2972 9	2.6	2.4	0.01	1.8	0
**Parameters**	**Condition**	**N_{cells}**	**n_{FBRs}**	**Mean**	**SD**	**SEM**	**Median**	**p-value (wrt Front)**
ηeff (Pa.s)	Front; Leader_S	66	15440	2045.5	2530. 4	20.3	1254.9	
	Mid; Leader_S	66	14413	2158.8	2191. 7	18.2	1532.4	7E-56
	Front; Leader_N	53	11624	2254.3	2627. 8	24.3	1348.2	
	Mid; Leader_N	53	15316	2341.3	2073. 3	16.7	1826.9	3E-84
	Front; All	119	27064	2135.1	2574. 7	15.6	1291.8	
	Mid; All	119	2972 9	2252.8	2133. 4	12.4	1680.9	7E-153

Biology Open • Supplementary information

Parameters	Condition	N_{cells}	n_{FBRs}	Mean	SD	SEM	Median	p-values (wrt Front)
γ (N/m^3)	Front; Leader_S	66	15440	0.9E9	1.2E9	1E7	0.5E9	
	Mid; Leader_S	66	14413	1E9	1.4E9	1.2E7	0.5E9	9E-4
	Front; Leader_N	53	11624	0.9E9	1.1E9	1E7	0.5E9	
	Mid; Leader_N	53	15316	1E9	1.4E9	1.2E7	0.5E9	8E-27
	Front; All	119	27064	0.9E9	1.2E9	0.7E7	0.4E9	
	Mid; All	119	29729	1E9	1.4E9	0.8E7	0.5E9	9E-26

Biology Open • Supplementary information

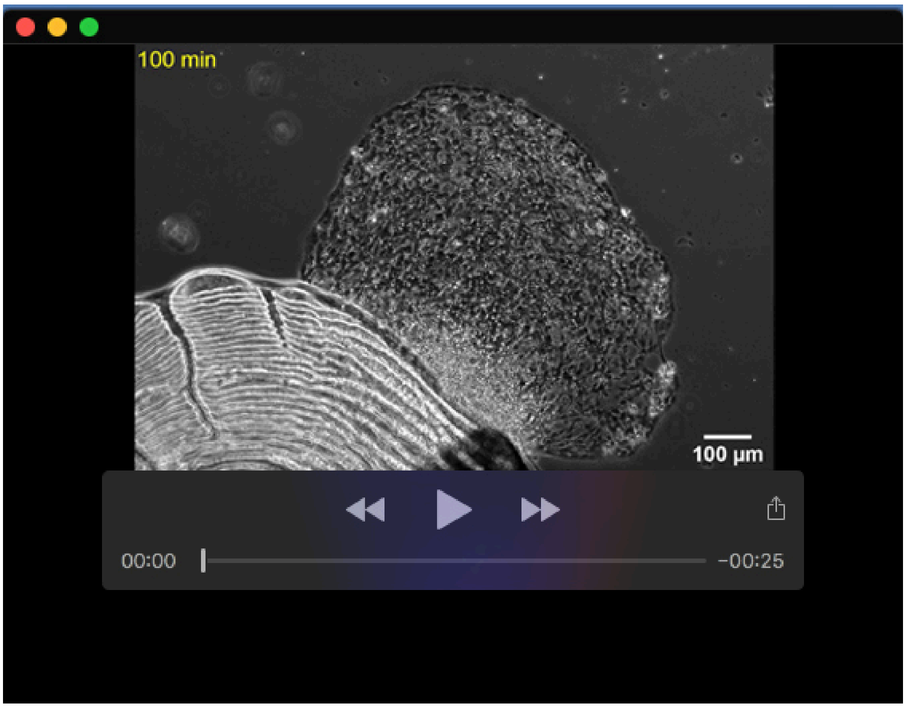

Movie 1. A representative movie of collective cell migration of cells emerging out of a fish scale. Phase-contrast images were acquired every 10 mins.

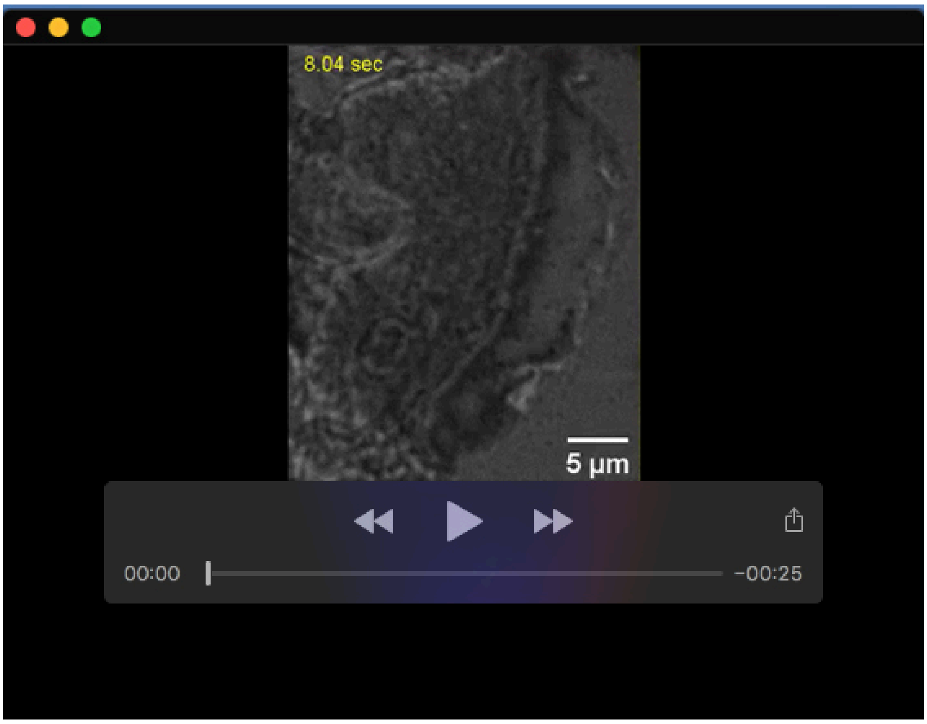

Movie 2. A representative movie of membrane fluctuations of a leader cell captured by IRM at 50 frames per sec acquisition rate.
